# Self-organization of active particles by quorum sensing rules

**DOI:** 10.1038/s41467-018-05675-7

**Published:** 2018-08-13

**Authors:** Tobias Bäuerle, Andreas Fischer, Thomas Speck, Clemens Bechinger

**Affiliations:** 1 Fachbereich Physik, Universität Konstanz, D-78464 Konstanz, Germany; 2 Institut für Physik, Johannes Gutenberg-Universität Mainz, D-55128 Mainz, Germany

## Abstract

Many microorganisms regulate their behaviour according to the density of neighbours. Such quorum sensing is important for the communication and organisation within bacterial populations. In contrast to living systems, where quorum sensing is determined by biochemical processes, the behaviour of synthetic active particles can be controlled by external fields. Accordingly they allow to investigate how variations of a density-dependent particle response affect their self-organisation. Here we experimentally and numerically demonstrate this concept using a suspension of light-activated active particles whose motility is individually controlled by an external feedback-loop, realised by a particle detection algorithm and a scanning laser system. Depending on how the particles’ motility varies with the density of neighbours, the system self-organises into aggregates with different size, density and shape. Since the individual particles’ response to their environment is almost freely programmable, this allows for detailed insights on how communication between motile particles affects their collective properties.

## Abstract (web summary)

Bacteria communicate and organize via quorum sensing which is determined by biochemical processes. Here the authors aim to reproduce this behaviour in a system of synthetic active particles whose motion is induced by an external beam which is in turn controlled by a feedback-loop which mimics quorum sensing.

## Introduction

One of the most intriguing properties of living matter is its ability to spontaneously organise from random into complex structures on different length scales such as biofilms^1^, swarms^2,3^ and flocks^4–7^. This requires communication between individual group members, which is typically realised by complex internal signal pathways. In the case of cells or bacterial colonies, communication can be achieved by extracellular signalling molecules which are produced, released and sensed by all group members^8,9^. Such biochemical communication, so-called quorum sensing, enables organisms to measure their local population density and to regulate their response accordingly (quorum sensing should be distinguished from chemotaxis describing the response of organisms in concentration gradients). The first example of quorum sensing was observed in the bioluminescent bacteria *Aliivibrio fischeri* which start to luminesce once their population density exceeds a certain density threshold^10^. By now, many other examples of quorum sensing have been found and it is considered to be a generic cell-to-cell communication mechanism, which is relevant, e.g., for the secretion of virulence factors^11^, biofilm formation^12^ and motility control^10,13^.

In contrast to living systems, where the organism’s response to a molecular concentration is determined by internal signal pathways, the motility of synthetic self-propelling active particles (APs) can be externally adjusted, e.g. by optical^14,15^, electrical^16,17^ or thermal^18,19^ fields. Experiments with such systems show a wealth of dynamical states ranging from living crystals^20^ to phase separation^20–22^ and swarming^16^ that can be controlled by geometry^23^ or boundary conditions^24^. In addition to suspensions with homogeneous motility, theoretical studies also considered APs whose motility and orientation changes upon variations of their local density^25–27^ or their own chemical concentration gradients^28^. Under such conditions, not only motility-induced phase separation but also the occurrence of moving clumps, lanes and asters has been observed.

Here we present an experimental realisation of an active suspension, whose individual particle motion varies depending on its neighbouring density. This is achieved using APs whose individual motility, i.e. magnitude of propulsion, is controlled by the intensity of an incident focused laser beam. In contrast, the propulsion direction, which is given by the particle orientation, remains unaffected by the laser illumination and undergoes free Brownian rotational diffusion. With an external feedback-loop which consists of a real-time optical particle detection algorithm which controls the position of a scanned laser beam, we are able to explore how a specific choice of a density-dependent particle motility affects the cooperative behaviour. With experiments, numerical simulations and theory we demonstrate that small variations on how particles change their motility in response to their environment can strongly affect their self-organisation.

## Results

### Experimental realisation

**Fig. 1:**
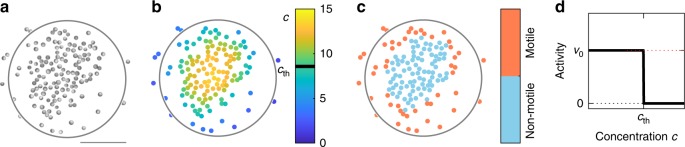
Collective behaviour of active particles with quorum-sensing rules. **a** Experimental snapshot of a two-dimensional suspension of carbon-coated APs with size *σ* = 4.4 μm (scale bar 50 μm). Upon laser illumination, particles self-propel as a result of a local demixing process in the binary solvent. **b** The colour code corresponds to the chemical concentration *c* which is attributed to each particle according to Eq. (1) for a decay length *λ* = 10*σ*. **c** Corresponding particle activity obtained by the concentration-dependent motility rule shown in **d** with concentration threshold $c_{{\mathrm{th}}} = 8.6\tilde c$. **d** Concentration-dependent particle motility with self-propulsion velocity $v_0$ below the threshold $c_{{\mathrm{th}}}$ and Brownian diffusion above

Active particles are made from silica spheres with diameter *σ* = 4.4 μm which are half-coated by a 30 nm carbon film and suspended in a critical mixture of water–lutidine several degrees below its lower demixing temperature *T*_c_. Upon laser illumination, the carbon caps are selectively heated above *T*_c_, which leads to local demixing of the solvent near the caps^14^. As a result of compositional flows within the solvent, the particles self-propel opposite to the cap with their speed determined by the incident laser intensity (Methods section). Because particles are separately illuminated by a scanned focused laser beam, we are able to dynamically assign each AP an individual propulsion velocity. It should be mentioned that the direction of the propulsion velocity is opposite to the cap orientation which is undergoing free Brownian rotational diffusion. Accordingly, in our experiments, only the magnitude but not the direction of motion is controlled externally. The entire suspension is contained in a thin sample cell with height 200 μm, where particles form a two-dimensional system due to gravitational forces. To avoid variations of the total particle density within our field of view, we have applied a lateral circular confinement with reflective boundary conditions and radius *R* = 65 μm (Fig. 1a). However, cluster formation by quorum-sensing rules, as reported here, is also observed using periodic boundary conditions (Methods section). In the following, we investigate a suspension with constant particle density *ρ*_0_ = 0.0092 μm^−2^ (corresponding $\simeq 15\%$ of close packing). To obtain steady-state conditions, we have allowed the system to evolve in time for at least 30 min before taking data. For further details of the experimental setup, (see Methods section).

Quorum sensing in living systems requires communication between individuals, which is achieved by the release of signalling molecules with production rate *γ*^29^. Because such molecules have a finite lifetime, each organism senses the molecular concentration

1 $$c_i\left( t \right) = \tilde c\mathop {\sum }\limits_{j \ne i} \frac{\sigma }{{r_{ij}\left( t \right)}}e^{ - \frac{{r_{ij}\left( t \right)}}{\lambda }}.$$

Here, $r_{ij} = | {\mathbf{r}}_i - {\mathbf{r}}_j |$ is the distance to the neighbour labelled *j*, *σ* the linear size of the individuals, and ${\lambda }= {\sqrt {D_{\mathrm{c}}\tau}}$ (with *D*_c_ the diffusion coefficient of the signalling molecules and *τ* their lifetime) a decay length, defining the range of the concentration profile and thus the distance over which particles communicate. The prefactor is given by $\tilde c = \gamma /\left( {4\pi D_{\rm{c}}\sigma } \right)$.

To externally introduce particle communication in a suspension of synthetic APs that are lacking the ability to release or detect such molecules, we first determine the APs’ positions in our sample at periodic time intervals. Next, we use Eq. (1) and calculate the hypothetical molecule concentration *c*_*i*_(*t*) at each AP (Fig. 1a, b). Because all concentrations in the following are given in units of $\tilde c$, our results are independent of the specific choice of *γ* and *D*_c_. The quasi-static chemical concentration profile suggested by Eq. (1) is valid in our system since the diffusive spreading of typical signalling molecules (e.g. N-acyl homoserine lactone (AHL), *D*_c_ ≈ 500 μm^2^ s^−1^ ^30^) is much faster than the overall motion of the APs.

To trigger a density-dependent motility response, we apply the following rule: when the concentration *c*_*i*_(*t*) ‘sensed’ by an AP exceeds a threshold, i.e. *c*_*i*_ > *c*_th_, it becomes non-motile (i.e. the laser illumination is set to zero and thus no self-propulsion occurs, *v* = 0), otherwise it is motile and propels with velocity *v* = *v*_0_ (Fig. 1c, d). Such a sharp threshold is in agreement with the conditions of many living systems exhibiting quorum sensing^10,13^. In the following, the propulsion velocity was set to *v*_0_ = 0.2 μm s^−1^ (Methods section). Due to diffusive and active motion, particles undergo configurational variations over time which lead to continual changes of the APs’ behaviour from motile to non-motile. Particle motilities are updated every 500 ms (Methods section). During this time interval, particles typically move by a distance <5% of their diameter. Note that the reduction of the motility update time by a factor of 100 yields identical results as confirmed by numerical simulations (Supplementary Figure 1).

**Fig. 2:**
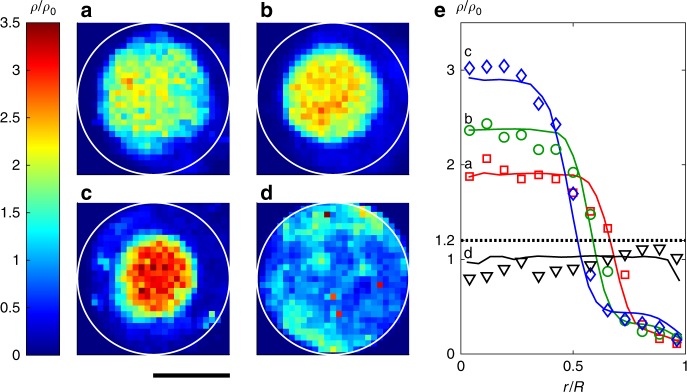
Density distribution of active particles. **a**–**d** Experimentally measured normalised particle density $\rho /\rho _0$ in the steady-state for concentration threshold values $c_{{\mathrm{th}}} = [5.9,7.3,8.6,\infty ]\tilde c$ (from **a** to **d**) and decay length *λ* = 10*σ*. Increasing the threshold value leads to more compact and smaller clusters. For permanently motile particles (*c*_th_ = ∞), no cluster formation is observed (scale bar 65 μm). **e** Comparison of the radial density distributions obtained from experiments (symbols) and simulations (lines). The dotted line corresponds to $\rho /\rho _0 = 1.2$ which defines the threshold for the definition of a cluster

For *c*_th_ = 0 (i.e. an entirely Brownian suspension), particles are homogeneously distributed. Increasing *c*_th_ leads to an inhomogeneous particle distribution and formation of clusters with growing density (Fig. 2a–c). In the following, clusters are defined by regions where the particle density *ρ* is 20% larger than *ρ*_0_. When *c*_th_ = ∞ (i.e. permanently motile APs), cluster formation again disappears. This is in contrast to APs with constant motility, where cluster formation is observed at much higher velocities and densities of APs^22,31^ (Supplementary Figure 2). This clearly demonstrates that here the organisation into densely packed regions is entirely due to the presence of quorum-sensing rules. The formation of clusters under such conditions is qualitatively understood as follows: isolated and motile particles approaching dense regions slow down; as a result, they become entirely diffusive since they sense super-threshold concentrations. This facilitates the aggregation of particles leading to cluster growth. Because particle self-propulsion is turned off when joining a cluster (in contrast to permanently motile particles), the density within clusters is rather loose as confirmed by the radial density profiles *ρ*(*r*), where *r* is measured relative to the clusters’ centre of mass (Fig. 2e). Such loose packing is in strong contrast to the closely packed (even crystalline) aggregates which are observed in dense suspensions of APs with constant motility^32^. Our experimentally measured density profiles are in excellent agreement with those obtained by numerical simulations (solid lines in Fig. 2e) of a simple model neglecting hydrodynamic interactions (Methods section).

### Numerical and analytical analysis

**Fig. 3:**
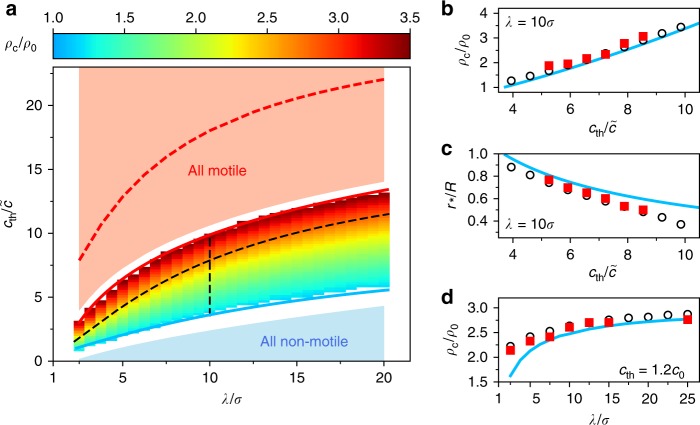
Phase behaviour. **a** Numerically determined phase diagram showing the dependence of the cluster density *ρ*_c_ on the threshold *c*_th_ and interaction length *λ*. Clustering occurs between the two solid lines (blue line: minimal threshold from theory, red line: limit of cluster stability), outside the system is homogeneous. Shaded areas indicate regions where all particles are either motile (red) or non-motile (blue). Red dashed line: theoretical limit where the cluster density reaches close packing. Black dashed lines correspond to paths along which experimental data were taken. **b** Cluster density *ρ*_c_ and **c** cluster radius r_*_ as a function of threshold *c*_th_ at *λ* = 10*σ* (vertical dashed line in **a**). **d** Cluster density as a function of decay length *λ*, holding the threshold *c*_th_ = 1.2*c*_0_ constant with respect to the reference concentration $c_0(\lambda ) = \frac{{\gamma \lambda \rho _0}}{{2D_c}}\left( {e^{ - \sigma /\lambda } - e^{ - R/\lambda }} \right)$ of a (hypothetical) homogeneous system (horizontal dashed line in **a**). In **b**–**d**, open symbols correspond to simulations, closed symbols to experiments and blue lines to theory. Experimental error bars (standard deviation) are comparable to the symbol size

To gain further insights into the influence of the quorum-sensing rule on the collective particle behaviour, we performed numerical simulations where the concentration threshold *c*_th_ and the decay length *λ* were systematically changed. In addition, we developed a mean-field theory from which analytical results can be obtained. It describes a passive, circular homogeneous cluster in coexistence with a motile gas due to the balance of a diffusive current away and an active current towards the cluster (Methods section). Our findings are summarised in Fig. 3a. In agreement with Fig. 2a–d, clustering occurs only within a well-defined range of concentration thresholds *c*_th_. Our numerical simulations predict cluster formation between the solid red and blue line (the latter also in agreement with the onset of cluster formation within the mean-field calculations). With increasing decay length *λ*, each particle senses a larger number of neighbours, i.e. a higher concentration (Eq. (1)). Accordingly, the maximal threshold *c*_th_ for which only non-motile particles are observed, becomes larger. The same trend also applies to the conditions where only motile particles exist. As can be seen, cluster formation occurs only within these limiting cases. With increasing *c*_th_ (*λ* = const.) the clusters are compressed, i.e. they become denser and smaller, as seen by the open symbols in Fig. 3b, c. This behaviour is in good agreement with our experiments (closed symbols) and qualitatively reproduced by the mean-field calculations (solid line) in which fluctuations and the excluded volume are neglected. As expected, the agreement with mean-field generally becomes better with increasing decay length since particles sense over larger distances and are thus less sensitive to fluctuations of the particle configuration (Fig. 3d and Supplementary Figure 3). The upper boundary for cluster formation suggested by mean-field theory is shown as a dashed red line and largely overestimates the limit where stable clusters are formed according to our experimental and numerical data. The reason is that clusters shrink as we increase the threshold, and clusters composed of a few particles become unstable with respect to fluctuations (which are neglected in mean-field). A small density fluctuation (e.g. because one particle leaves the cluster) might lead to a drop of the chemical concentration below the threshold for other particles in the cluster, which then become motile and also leave the cluster. For small clusters, this positive feedback between density and motility fluctuations is sufficient to dissolve the entire cluster while larger clusters remain stable. In the simulations, we observe that clusters with less than *N*_p_ ≈ 65 passive particles spontaneously dissolve (and also re-form) independent of *λ* (Supplementary Figure 4). This effect lowers the upper boundary in Fig. 3a to much smaller concentration thresholds. Larger global densities stabilise clusters at larger thresholds and move the upper boundary closer to the theoretical limit of closely packed clusters (Supplementary Figure 5). The actual number below which clusters dissolve depends on the system size (Supplementary Figure 6).

**Fig. 4:**
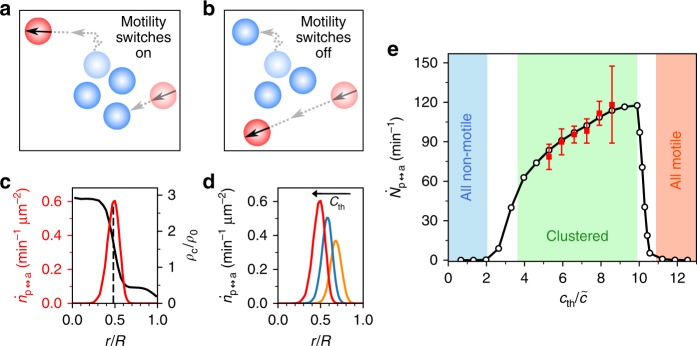
Cluster formation by motility switching. **a** Schematic illustration of the influence of motility switches. An active particle (red) is approaching a cluster of three passive particles (blue) and becomes passive which favours joining the cluster. On the opposite, a passive particle slightly diffusing away from a cluster becomes active which facilitates leaving it. **b** Same situation without motility changes. **c** Numerically obtained radial density profile *ρ*(*r*) and motility change density $\dot n_{\mathrm{p} \leftrightarrow \mathrm{a}}(r)$ for *λ* = 10*σ* and a concentration threshold *c*_th_ = 8.6$\tilde c$. Motility changes are localised at the interfacial region, the centre of the interface (dashed line) coincides with the maximum of $\dot n_{\mathrm{p} \leftrightarrow \mathrm{a}}(r)$. **d** Motility change density $\dot n_{\mathrm{p} \leftrightarrow \mathrm{a}}(r)$ at *λ* = 10*σ* and *c*_th_ = [5.9,7.3,8.6]$\tilde c$. With increasing *c*_th_, the distribution increases in height and is shifted to smaller *r* (as the cluster size decreases). **e** Total rate of motility changes $\dot N_{\mathrm{p} \leftrightarrow \mathrm{a}}$ versus *c*_th_ for *λ* = 10*σ* from simulations (open) and experiments (closed symbols). Error bars correspond to the standard deviation of different measurements

Our experiments and simulations suggest that cluster formation resulting from quorum-sensing rules requires not only the coexistence of motile and non-motile particles (Fig. 3a), but also the ability to change their motility (Fig. 4a, b). To investigate the importance of such motility changes in more detail, we performed simulations with mixtures of motile and non-motile particles but without the possibility of motility changes. Independent of the mixing ratio, no clustering is observed for our packing fraction (data not shown).

In order to quantify the motility changes, we introduce the motility change density $\dot n_{{\mathrm{p}} \leftrightarrow {\mathrm{a}}}(r)$, which is the number of motile-passive change events within a circular ring at distance *r* from the centre of the cluster per time and area. Figure 4c compares how $\dot n_{{\mathrm{p}} \leftrightarrow {\mathrm{a}}}(r)$ and the particle density profile *ρ*(*r*) depend on the radial position within a cluster for *λ* = 10*σ* and *c*_th_ = 8.6$\tilde c$. The rate $\dot n_{{\mathrm{p}} \leftrightarrow {\mathrm{a}}}(r)$ becomes largest at the interface between the dense cluster and the (dilute) gas because configurational changes of the APs are most pronounced near the interface. Accordingly, the distributions are shifted to smaller radii (Fig. 4d) when the cluster size becomes smaller by increasing the sensing threshold *c*_th_ (cf. Figure 2a–d). In Fig. 4e, we have plotted the total rate of motility changes $\dot N_{{\mathrm{p}} \leftrightarrow {\mathrm{a}}}$ (density $\dot n_{{\mathrm{p}} \leftrightarrow {\mathrm{a}}}(r)$ integrated over the whole area of the system) vs. *c*_th_. Obviously, induced cluster formation requires a minimal rate of motility changes. Starting from a homogeneous particle distribution (*c*_th_ = 0), a growing threshold *c*_th_ leads to clusters with increasing density (cf. Fig. 3a). Accordingly, the deviation of the particle distribution compared to thermal equilibrium increases. Sustaining such non-equilibrium conditions requires an increasing amount of motility control, i.e. switching rate $\dot N_{{\mathrm{p}} \leftrightarrow {\mathrm{a}}}$. As the clusters become unstable with respect to fluctuations, the switching rate drops and becomes again zero when all particles remain active. The behaviour shown in Fig. 4e is robust with respect to changing of *λ* (Supplementary Figure 7) and thus demonstrates that the dynamics of motility changes plays an important role for cluster formation induced by quorum-sensing rules.

**Fig. 5:**
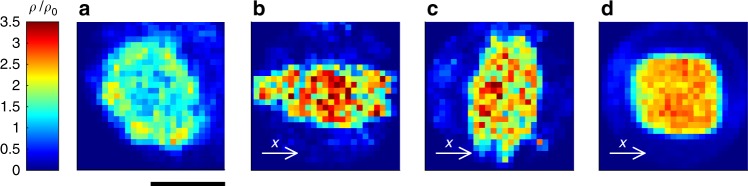
Experimentally measured structural changes due to motility response variations. **a** ‘Ring’: when the local density *c* exceeds a second threshold *c*_th,2_ > *c*_th_, the swimming velocity is set to *v*_0_ again. This reduces the density in the centre of the cluster (scale bar 65 μm). **b** ‘Ellipse’: introducing an angle-dependent term *f*(Θ) = cos^4^(Θ). **c** Changing *f*(Θ) to *f*(Θ) = sin^4^(Θ) leads to rotation of the ellipse by 90°. **d** ‘Square’: doubling the frequency of the angle-dependent term to *f*(Θ) = cos^4^(2⋅Θ) leads to a square-shaped cluster. In (**b**–**d**) the arrow shows the direction of the *x*-axis

We have also studied how the collective behaviour changes upon further variations of the particles’ response to their environment. Figure 5a shows a cluster that is formed by introducing a second concentration threshold *c*_th,2_ > *c*_th_ above which particles recover their motility^27^. This leads to enhanced active particle motion near the cluster centre and preferential escape from this region. As a result, the particle density near the centre decreases which leads to a ‘ring’-like structure.

We also considered the situation where the concentration profile of signalling molecules becomes non-isotropic, e.g. due to thermophoretic forces induced by external temperature gradients. To account for the angle-dependence of the concentration profile around each particle *j*, we have modified Eq. (1) by an angle-dependent function *f*(Θ) to

2 $$c_i\left( t \right) = \tilde c\mathop {\sum }\limits_{i \ne j} \frac{\sigma }{{r_{ij}\left( t \right)}}e^{ - \frac{{r_{ij}\left( t \right)}}{\lambda }}f\left( {{{\mathrm{\Theta }}}_{ij}} \right)$$

where Θ_*ij*_ denotes the angle between the connecting vector of particles *i* and *j* and the *x*-axis. For *f*(Θ) = cos^4^(Θ) we find an elliptically elongated shape, whose axis is rotated by 90° for *f*(Θ) = sin^4^(Θ) (Fig. 5b, c). Choosing *f*(Θ) = cos^4^(2⋅Θ) we obtain an almost quadratically shaped cluster as shown in Fig. 5d.

## Discussion

We have demonstrated the collective behaviour of synthetic particles which interact via quorum sensing rules. Contrary to living systems, where the interpretation of signalling molecules can be rather complex^33^, in our experiments well-defined perception-response relations are imposed externally by a feedback-loop. Because our approach does not rely on specific particle interactions, this enables us to freely vary not only the type of stimuli but also how particles respond to them. In addition to a density-dependent incentive which controls the particles’ motility, other types of particle responses including active torques^34^ but also a time-delay between a stimulus and the particles’ response and non-reciprocal interaction rules can be realised. Finally, since our experiments are performed at low Reynolds number akin to the surroundings of bacteria, comparison of the collective behaviour of synthetic with living systems may allow to unveil what type and over what distances information must be exchanged to initiate collective behaviour.

## Methods

### Experimental details

In our experiments we use silica particles with diameter *σ* = 4.4 μm and a 30-nm-thick carbon film on one hemisphere, which are suspended in a critical mixture of water–lutidine at temperature *T* = 25 °C. Particles are individually illuminated with a scanned laser beam (beam waist *w* = 5 μm) aiming at the centre of each particle. Under such conditions, particles self-propel with velocity *v* opposite to the orientation of the capped hemisphere which is undergoing rotational Brownian diffusion. It is important to notice that this reorientation is not altered by the illumination.

The translational and rotational diffusion coefficients have been determined to *D*_0,exp_ = (0.0208 ± 0.0012) μm^2^ s^−1^ and *D*_R,exp_ = (112.6 ± 26.1)^−1^ s^−1^ by evaluating the translational mean-square displacement in a dilute system^35^. While *D*_R,exp_ agrees well with the theoretical value, *D*_0,exp_ is about 50% below the bulk Stokes–Einstein value. The reduction is due to the enhanced viscous friction near a surface^36^ and in agreement with previous studies^14^.

To avoid changes in the particle density during experiments, we employ reflective boundary conditions at the edge of a circular confinement with radius *R* = 65 μm by the application of an active torque which leads to a particle reorientation^14,34^. This is accomplished by displacing the illuminating laser beam relative to the particle centre by ≈2.6 μm resulting in a local intensity gradient. This causes an effective torque and thus particle reorientation. Such torques are applied to particles when leaving the circular confinement until their swimming direction points towards the confinement centre. To reduce variations of the calculated concentration by particles leaving and re-entering the confinement, we take all particles into account which leave the confinement by <10 μm. The confinement contains on average 122 particles, corresponding to a density of *ρ*_0_ = 0.0092 μm^−2^.

### Experimental realisation of feedback-controlled particle motility

The propulsion velocity of the particles is controlled independently from each other by a feedback loop. A video camera acquires images with a repetition rate of 2 Hz which are then evaluated on a computer by a real-time particle detection algorithm to determine the particle positions. With an acousto-optical deflector (AOD) a laser beam, with beam waist *w* = 5 μm, is consecutively directed to the prior determined particle positions and each particle is illuminated for a period of 8 μs which is repeated every 4 ms. Since the remixing timescale of the binary mixture is on the order of 100 ms^14^, the repetition is fast enough to produce stable particle self-propulsion conditions. With this approach, the motilities of up to 400 particles can be controlled independently.

**Fig. 6:**
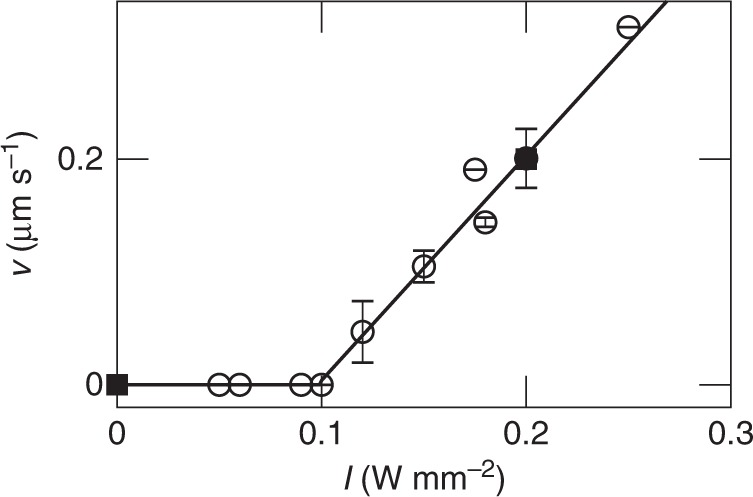
Swimming velocity. Experimentally observed particle velocities (open symbols) depending on the intensity of the laser illumination. Above an intensity of *I* = 0.1 W mm^−2^ the particle starts to self-propel by demixing the solvent around the carbon-coated hemisphere and the velocity *v* then depends linearly on the intensity *I*. In our experiments we use *I* = 0.2 W mm^−2^ for motile particles resulting in *v* = 0.2 μm s^−1^, and no illumination for non-motile particles (squares). Error bars correspond to the standard deviation of different measurements

The time-averaged illumination intensity of each particle is set to either *I* = 0.2 W mm^−2^ (resulting in a propulsion velocity *v*_0_ = 0.2 μm s^−1^) or to *I* = 0 (diffusive motion) (Fig. 6). Particle configurations are updated every 500 ms, typical positional changes during this time interval are below 5% of the particle diameter. Therefore, the feedback-loop can be considered as quasi-instantaneous.

### Illumination intensity corrections

To avoid velocity changes due to the overlap of the illuminating Gaussian beams of neighbouring particles, we have additionally adjusted the laser intensity depending on the relative particle positions. The intensity profile of the laser beam illuminating particle *i* is given by

3 $$I_i(r) = I_{0,i} \cdot e^{ - \frac{{2r^2}}{{w^2}}},$$

where *w* = 5 μm is the beam waist, *I*_0,*i*_ its intensity and *r* the radial distance. Accordingly, when particles (diameter 4.4 μm) get close to each other, they will also receive light from the beams centred on their neighbours. This leads to an increased intensity at the centre of particle *i* by

4 $${{\mathrm{\Delta }}}I_i = \mathop {\sum }\limits_{i \ne j} I_{0,j} \cdot e^{ - \frac{{2r_{ij}^2}}{{w^2}}},$$

with *r*_*ij*_ the distance between particles *i* and *j*. To avoid such configuration-dependent variations in the effective illuminating intensity (and thus of the propulsion velocity), we corrected the illuminating intensity by reducing the intensity of beam *i* to

5 $$\hat I_{0,i} = \frac{{I_{0,i}}}{{(1 + {{\mathrm{\Delta }}}I_i)^{1.4}}}.$$

**Fig. 7:**
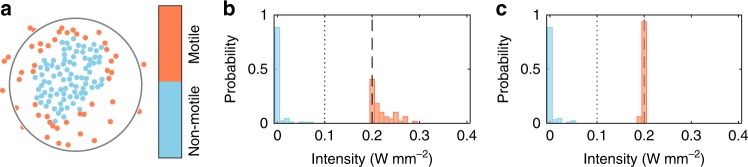
Adjustment of the illumination intensity. **a** Particle configuration taken from Fig. 1a–c with assigned particle activity. Motile particles are supposed to be illuminated with intensity *I* = 0.2 W mm^−2^ (dashed line in **b**, **c**) and non-motile ones with intensity *I* < 0.1 W mm^−2^ (onset of propulsion, dotted line in **b**, **c**). **b** Without corrections of the illumination intensity, the majority of motile particles (red) is illuminated too strong. **c** Using the intensity adjustment according to Eq. (5), all motile particles are illuminated with the allocated intensity

For computational reasons, we only consider particles *j* with *r*_*ij*_ < 2*w* in the calculation of Δ*I*_*i*_. Using this empirical relationship, the illumination integrated over each particle becomes independent of the positional configuration. To demonstrate the validity of Eq. (5), we have numerically tested this for a huge number of arbitrary particle configurations including situations with and without applied quorum sensing interaction. Figure 7 shows an example (corresponding to the particle configuration in Fig. 1a–c) with motile and non-motile APs. We have considered an illumination intensity of non-motile and motile particles of *I* = 0 and *I* = 0.2 W mm^−2^, respectively. Without the correction discussed, we obtain a bimodal illumination intensity distribution of the particles as shown in Fig. 7b. Obviously, some of the motile particles receive up to 0.3 W mm^−2^, i.e. 50% more than the nominal illumination intensity. After application of the correction procedure, this unwanted effect is almost completely suppressed (Fig. 7c). Note, that the correction is less effective for passive particles. However, due to the presence of a minimal intensity to initiate active motion (dotted line, *I* = 0.1 W mm^−2^), this will not change the behaviour of non-motile particles.

### Simulations

In our numerical simulations, we integrate the coupled equations of motion (assuming overdamped dynamics and neglecting hydrodynamic interactions)

6 $$\frac{\partial }{{\partial t}}{\mathbf{r}}_i = v\left( {c_i} \right){\mathbf{e}}_i - \frac{{D_0}}{{k_{\rm{B}}T}}\nabla_{\!i} U\left( {\left\{ {{\mathbf{r}}_j} \right\}} \right) + {\mathbf{\xi }}_i$$

for *N* particles at positions **r**_*i*_. A particle is propelled along its orientation vector **e**_*i*_ with velocity *v*_0_ = 0.2 μm s^−1^ if it senses a concentration *c*_*i*_ < *c*_th_ (with *c*_*i*_ determined by Eq. (1)), for *c*_*i*_ > *c*_th_ the propulsion is set to zero and the particle is non-motile. The particle orientations undergo rotational diffusion with rotational diffusion coefficient *D*_R_ = (1/120) s^−1^. Translational diffusion is modelled by the random force **ξ**_*i*_ with zero mean and variance <**ξ**_*i*_(*t*)**ξ**_*j*_(*t*′) > = 2*D*_0_*δ*_*ij*_*δ*(*t* − *t*′) with translational diffusion coefficient* D*_0_ = 0.02 μm^2^ s^−1^. We model steric particle interactions via the repulsive Weeks–Chandler–Andersen potential

7 $$U(r_{ij}) = 4{\it{\epsilon }}\left[ {\left( {\frac{{\sigma _{{{\mathrm{wca}}}}}}{{r_{ij}}}} \right)^{12} - \left( {\frac{{\sigma _{{{\mathrm{wca}}}}}}{{r_{ij}}}} \right)^6 + \frac{1}{4}} \right]$$

cut off at *r*_cut_ = 2^1/6^*σ*_wca_. We set ${\it{\epsilon }} = 100\,k_{\rm{B}}T$ and *σ*_wca_ = 3.98 μm, which implies an effective (Barker-Henderson) particle diameter *σ* = 4.4 μm^37^. The particles are randomly initialised and the equations of motion are integrated with time step Δ*t* = 40 ms. The concentrations *c*_*i*_ are updated every time step. To compare the rate of motility changes to the experiment, motility changes are recorded every 480 ms.

**Fig. 8:**
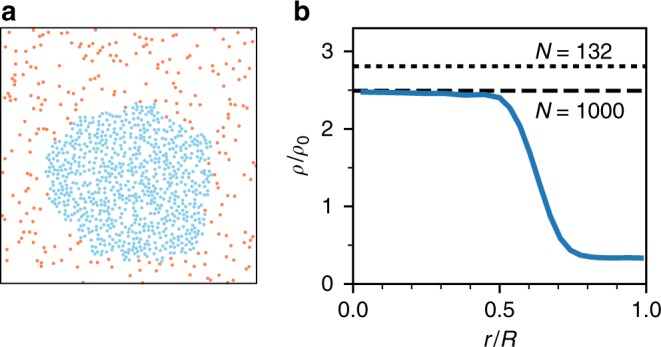
Cluster formation with periodic boundary conditions. **a** Snapshot of a simulation with *N* = 1000 particles with density *ρ*_0_ = 0.0075 μm^−2^ (*λ* = 5*σ*, *c*_th_ = 5.5$\tilde c$). As in the confined system, we observe the formation of a cluster of non-motile particles (blue) surrounded by a dilute gas of motile particles (red). **b** Blue: Corresponding radial density profile *ρ*(*r*)/*ρ*_0_ (with respect to the particles’ centre of mass) with *R* half the box length. The cluster density is the same as for a circularly confined system with *N* = 1000 particles at the same density (dashed line). For the smaller system with *N* = 132, the cluster density is slightly higher (dotted line). The reason is that due to the smaller confinement, quorum concentrations are lower and thus the particles have to form a denser cluster to overcome the threshold

We have performed two types of simulations: employing periodic boundary conditions (shown in Fig. 8) and modelling the experimental system through a circular confinement with *N* = 132. For the latter, instead of applying a torque to particles reaching the boundary, in the simulations their orientation vectors are instantaneously reoriented towards the centre of the confinement with *R* = (65 + 10) μm. As shown in Supplementary Figure 1, our results are robust with respect to changing the time between motility updates.

### Analytical theory

Neglecting the excluded volume of particles, some insights can be obtained from a simplified mean-field theory of our model extending a previous approach^27^. The evolution of an ensemble of APs with joint probability *ψ*(**r**, *φ*, *t*) is governed by

8 $$\partial _t\psi = - \nabla \cdot \left[ {v{\mathbf{e}} - D_0\nabla } \right]\psi + D_{\mathrm{R}}\partial _\varphi ^2\psi$$

with scalar speed *v*(*c*) and orientation **e** = (cos *φ*, sin *φ*)^*T*^. We strongly simplify the experimental situation and assume that APs interact only through the chemical concentration profile *c*(**r**) generated by the APs, which we assume to adapt instantaneously to a change of particle positions (exploiting the huge difference between colloidal, *D*_0_, and molecular, *D*_c_, diffusion coefficients). This is the standard model of active Brownian particles (ABPs) extended by interactions through an additional scalar field *c*(**r**).

It is sufficient to consider only the first two moments

9 $$\rho \left( {{\mathbf{r}},t} \right) = \mathop {\smallint }\limits_0^{2\pi } {\mathrm{d}}\varphi \,\psi ({\mathbf{r}},\varphi ,t)\,,\quad {\mathbf{p}}\left( {{\mathbf{r}},t} \right) = \mathop {\smallint }\limits_0^{2\pi } {\mathrm{d}}\varphi {\mathbf{e}}\,\psi \left( {{\mathbf{r}},\varphi ,t} \right).$$

We consider stationary profiles with rotational symmetry and vanishing angular polarisation. Switching to polar coordinates with distance *r* from the origin (the centre of the cluster), we obtain

10 $$0 = vp - D_0\frac{{\partial \rho }}{{\partial r}},\quad 0 = - \frac{v}{2}\frac{{\partial \rho }}{{\partial r}} + D_0\left( {\nabla ^2p - \frac{p}{{r^2}}} \right) - D_{\mathrm{R}}p$$

with density profile *ρ*(*r*) and radial polarisation *p*(*r*). The first equation expresses the balance between active and diffusive particle currents. Eliminating the density, we obtain the Bessel differential equation

11 $$0 = p\prime\prime + \frac{{p\prime }}{r} - \left( {\frac{1}{{r^2}} + \frac{1}{{\xi ^2}}} \right)p$$

for the radial polarisation, the solution of which is a function of *r*/*ξ* with the length scale $\xi = \left( {\frac{{v^2}}{{2D_0^2}} + \frac{{D_R}}{{D_0}}} \right)^{ - \frac{1}{2}}$. We have two solutions, one for the inner passive region with speed *v* = 0 and radius r_*_, the other for the active gas with speed *v* = *v*_0_. In the inner region, from Eq. (10) we find that the density gradient is zero and thus the density *ρ* = *ρ*_c_ is constant with vanishing polarisation. In the outer region *r* > r_*_, the polarisation decays with *p*(*r*) = −*bK*_1_(*r*/*ξ*) with integration constant *b* and modified Bessel function of the second kind *K*_*n*_(*x*). Through integration, we obtain the density profile

12 $$\rho (r) = \rho _c + \frac{{vb\xi }}{{D_0}}\left[ {K_0(r/\xi ) - K_0(r_ \star /\xi )} \right]$$

for *r* > r_*_. Equation (10) also implies that the density is continuous at r_*_ and thus the polarisation has to jump. The jump condition and conservation of the total density allow to determine *ρ*_c_ and *b*.

The remaining unknown r_*_ is determined by the concentration threshold. The concentration profile reads

13 $$c\left( {\mathbf{r}} \right) = {\int} {{\mathrm{d}}{\mathbf{r}}\prime } \rho \left( {{\mathbf{r}}\prime } \right)u\left( {\left| {{\mathbf{r}} - {\mathbf{r}}\prime } \right|} \right),\quad u(r) = \tilde c\frac{{e^{ - r/\lambda }}}{{r/\sigma }}$$

with condition *c*(r_*_) = *c*_th_, which yields r_*_. We solve the resulting system of equations iteratively to obtain the cluster size r_*_ and density *ρ*_c_ for given threshold *c*_th_ and interaction range *λ* (Fig. 3).

### Data availability

The experimental and numerical data that support the findings of this study are available from the corresponding author upon reasonable request.

## Electronic supplementary material

Supplementary Info
