# Supplementary material for: Self-organization of active particles by quorum sensing rules
Source: Nat Commun. 2018 Aug 13;9:3232. doi: 10.1038/s41467-018-05675-7 (PMC6089911; doi:10.1038/s41467-018-05675-7)
Supplement: Supplementary file 1 — Supplementary Info [file 41467_2018_5675_MOESM1_ESM.pdf]

Supplementary Information

Self-Organization of Active Particles by Quorum Sensing Rules

Bäuerle et al.

Supplementary Figures

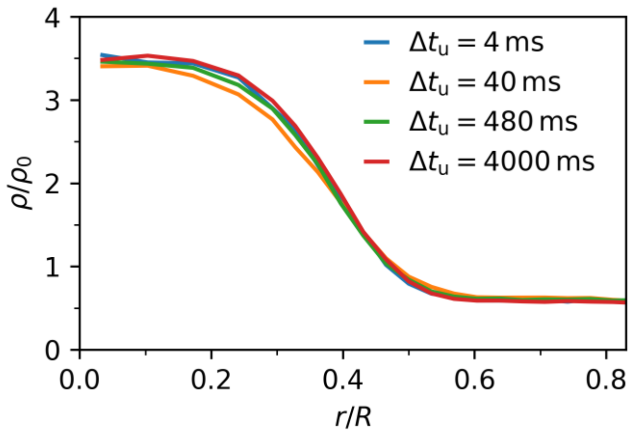

Supplementary Figure 1 Motility update frequency. Radial density profiles for different motility update intervals Δt_u (at $\lambda = 10\sigma$, $c_\mathrm{th} = 9.9\tilde{c}$). There is no visible difference varying the interval from 4 ms to 4000 ms (40 ms was used in the simulations in the main text, 500 ms in the experiment).

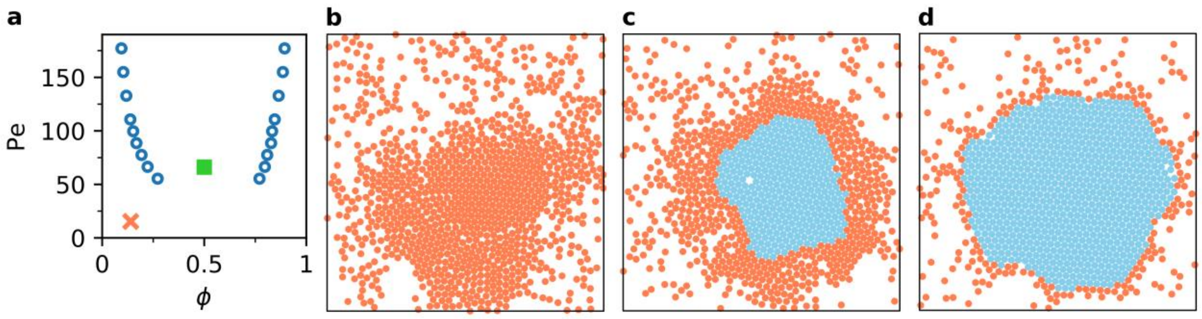

Supplementary Figure 2 Motility induced phase separation. a Circles: Numerically determined coexisting densities for plain active Brownian particles (ABPs, data taken from Ref.[1]) with packing fraction ϕ and Peclet number Pe $= 3v_0/(\sigma D_\mathrm{R})$. Red cross: experimental conditions of the present study, no clustering occurs for constant motility. **b,c,d** Simulation snapshots of a system of $N = 1000$ particles with periodic boundary conditions. Simulations are performed within the coexistence region at propulsion speed $4v_0 = 0.8$ µm s^{-1} and density $4.4\rho_0$ (green square in **a**). **b** Plain ABPs with all particles active (red). **c,d** With quorum sensing using $\lambda = 2.5\sigma$ and **c** $c_\mathrm{th} = 13.5\tilde{c}$, **d** $c_\mathrm{th} = 10\tilde{c}$. An inner core of crystalline closely packed passive particles (blue) emerges, which grows as the threshold is decreased. At the same time, the dense halo of active particles shrinks.

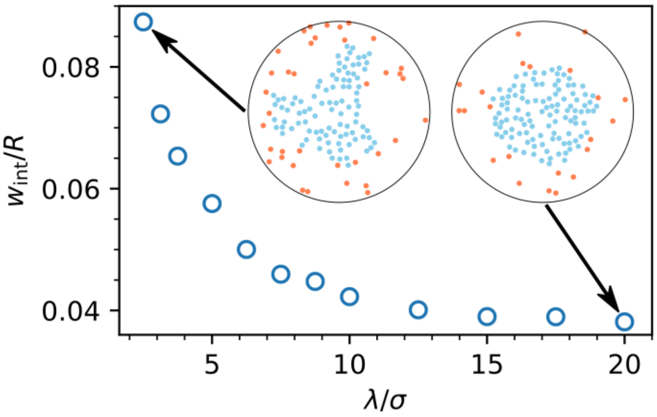

Supplementary Figure 3 Interfacial width of clusters. Interfacial width w_{int} versus interaction length λ. w_{int} is determined from fitting the obtained density profiles $\rho(r)$ with $\rho(r) = \frac{\rho_c+\rho_g}{2} - \frac{\rho_c-\rho_g}{2}\tanh\left(\frac{r-r_*}{2w_{\mathrm{int}}}\right)$, where ρ_c and ρ_g are the density of the cluster and the density of the active gas, respectively, and r_* the centre of the surface. For all λ the threshold c_{th} was chosen such that the clusters consists of $< N_p > \approx 100$ particles. Shape fluctuations are enhanced considerably for smaller λ (as shown by the simulation snapshots for $\lambda = 2.5\sigma, 20\sigma$), leading to an increased interfacial width.

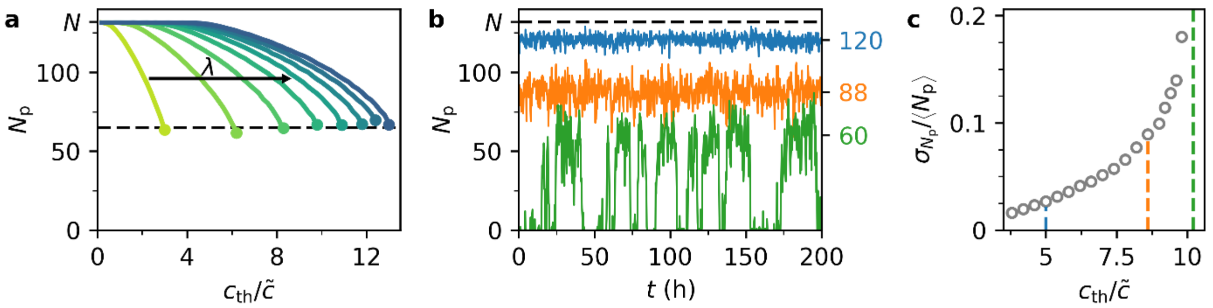

Supplementary Figure 4 Fluctuations of passive particles and cluster stability. a Average number of passive particles $< N_p >$ from simulations of a system with $N = 132$ particles as a function of threshold c_{th} for $\lambda = [2.5,5,7.5,10,12.5,15,17.5,20]\sigma$ (from left to right). For each λ value the disk indicates the maximum value of c_{th} up to which we observe stable clusters. Independent of λ, the circles lie very well on the $N_p = 65$ (dashed) line. **b** Time series $N_p(t)$ at $\lambda = 10\sigma$ for concentration thresholds $c_{\mathrm{th}} = [5.0,8.6,10.2]\tilde{c}$ (from top to bottom) with averages indicated on the right (for $c_{\mathrm{th}} = 10.2\tilde{c}$ the time frames in which no cluster existed were excluded from the averaging). **c** Standard deviation of $N_p(t)$ divided by the average, $\sigma_{N_p}/< N_p >$, which rises with c_{th} (only shown in the regime where stable clusters exist). Dashed lines indicate the threshold values employed in **b**.

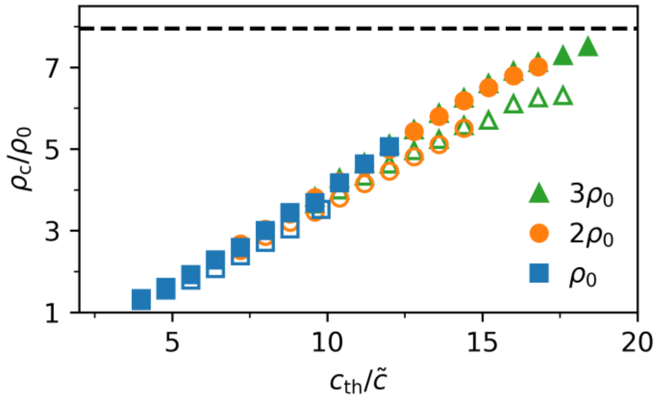

Supplementary Figure 5 Clustering at higher propulsion speed and density. Cluster density ρ_c versus threshold c_{th} for different global densities (colours) and propulsion speeds $v_0 = 0.2$ µm s^{-1} (empty symbols) and $3v_0 = 0.6$ µm s^{-1} (filled symbols) with $\lambda = 10\sigma, N = 132$ fixed. The dashed line indicates the close packing density $\rho \approx 8.0\rho_0$. Higher speed leads to higher cluster densities at fixed c_{th}. The upper threshold boundary for cluster stability is raised for increased speed and global density.

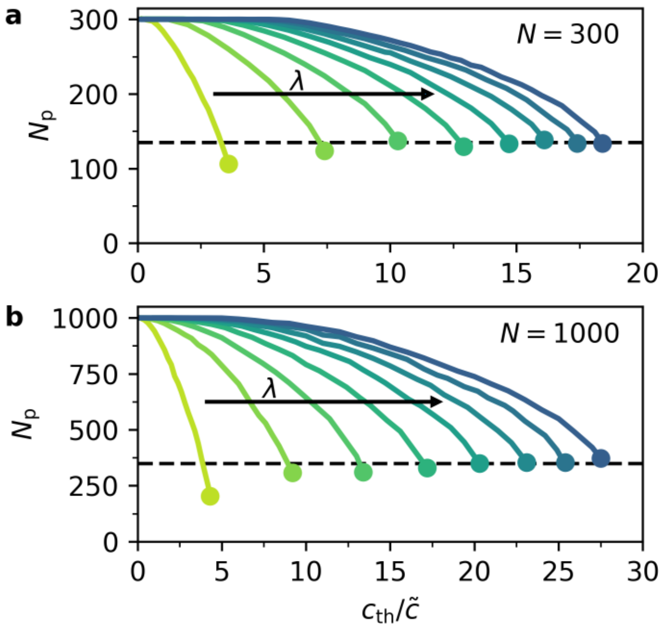

Supplementary Figure 6 Finite size behaviour. Number of passive particles N_p versus threshold c_{th} for interaction lengths $\lambda = [2.5,5,7.5,10,12.5,15,17.5,20]\sigma$ (from left to right) for the circularly confined system at fixed global density ρ_0 and particle numbers **a** $N = 300$, **b** $N = 1000$. The dashed lines indicate the minimal number of passive particles up to which stable cluster are observed ($N_p \approx 135$ for $N = 300$ and $N_p \approx 350$ for $N = 1000$). For small λ, the minimal particle number is slightly lower.

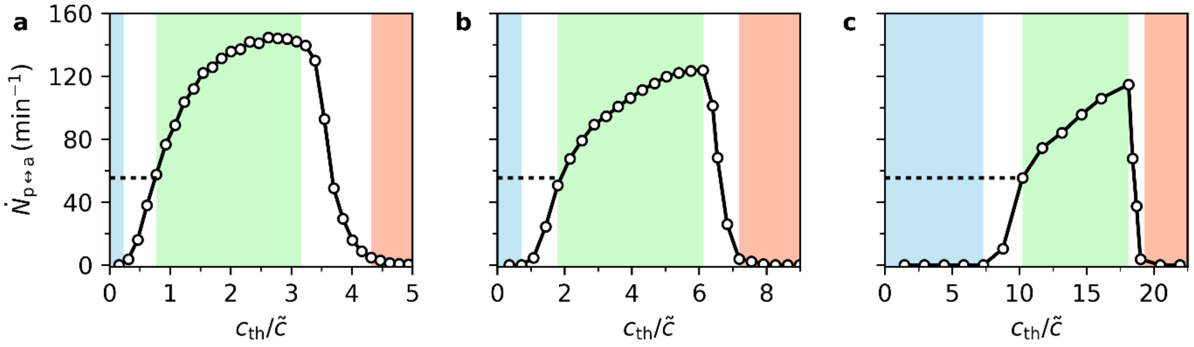

Supplementary Figure 7 Motility change rate dependence on λ. Total rate of motility changes $\dot{N}_{\mathrm{p}\leftrightarrow\mathrm{a}}$ versus concentration threshold c_{th} for three different values of λ (**a** $\lambda = 2.5\sigma$, **b** $\lambda = 5\sigma$, **c** $\lambda = \infty$) determined from simulations. Within the green region a stable cluster exists, whereas in the blue and red areas, we observe only non-motile and motile particles, respectively. The minimal rate for a stable cluster to exist when increasing c_{th} appears to be independent of λ (dashed lines). The rate of motility changes is generally higher for smaller λ and the drop of the rate above the region of stable clusters is less abrupt, because under such conditions fluctuations are more prominent.

Supplementary References

1. Siebert, J. T., Letz, J., Speck, T. & Virnau, P. Phase behavior of active Brownian disks, spheres, and dimers. *Soft Matter* **13,** 1020–1026 (2017).
